# New Spectrophotometric Method for Quantitative Characterization of Density-Driven Convective Instability

**DOI:** 10.3390/polym13040661

**Published:** 2021-02-23

**Authors:** Ying Teng, Pengfei Wang, Lanlan Jiang, Yu Liu, Yang Wei

**Affiliations:** 1 Institute for Advanced Study, Shenzhen University, Shenzhen 518060, China; tengying@szu.edu.cn; 2 College of Physics and Optoelectronic Engineering, Shenzhen University, Shenzhen 518060, China; 3 School of Earth and Space Sciences, University of Science and Technology of China, Hefei 230026, China; wangpf6@sustech.edu.cn; 4 Department of Physics, Southern University of Science and Technology, Shenzhen 518055, China; 5 Key Laboratory of Ocean Energy Utilization and Energy Conservation of Ministry of Education, Dalian University of Technology, Dalian 116024, China; lanlan@dlut.edu.cn

**Keywords:** convective dissolution, dissolved CO_2_ mass measurements, spectrophotometric method, CO_2_ storage in saline formations

## Abstract

CO_2_ convective dissolution has been regarded as one of the fundamental mechanisms to accelerate the mass transfer of CO_2_ into brine. We present a new spectrophotometric method to characterize the convective instability and measure the dissolved CO_2_ mass, which enables the real-time quantitative visualization of CO_2_/brine transport mechanisms. Successive images were captured to identify the finger development regimes, and the convection morphologies were analyzed by the fingers length and affected area. CO_2_ solubility was experimentally studied, and the results are in agreement with the theoretical calculations. CO_2_ mass transfer flux was investigated as the Sherwood number changed. The increase in salinity and temperature has a negative effect on CO_2_ dissolution; here, numerical simulation and experimental phenomena are qualitatively consistent. In general, these findings confirm the feasibility of the method and improve the understanding of the physical process of CO_2_ convective dissolution, which can help assess the CO_2_ solubility trapping mass.

## 1. Introduction

The Earth’s climate changes as a result of the increase of greenhouse gas emissions, particularly emissions of carbon dioxide (CO_2_) from burning fossil fuels [1]. Carbon capture and storage (CCS) technology is often considered as a cost-effective solution to mitigate climate change [2]. Saline aquifers have been recognized as promising storage sites due to their large capacity and widespread distribution across the world [3]. One fundamental physical phenomenon related to CO_2_ geological storage in saline aquifers is CO_2_ dissolution into brine [4]. Once CO_2_ is injected in brine-bearing formations, the less dense CO_2_ rises upwards until it is confined by an impermeable caprock where it spreads laterally beneath; then, it starts to dissolve into the underlying brine [5]. CO_2_-dissolved brine is 0.1–1% denser than the original brine [6]. Thus, a gravitationally unstable system develops with CO_2_-dissolved brine overlying the original brine, and this leads to density-driven convection and triggers instabilities.

CO_2_ dissolution in brine causes density-driven convection, which is a key mechanism for the efficient trapping of CO_2_. CO_2_ convective dissolution accelerates the CO_2_ mass transfer rate and reduces the risk of leakage, which is favorable for long-term sequestration [7]. Although there are some CCS pilot projects distributed in different parts of the world, there is still a lack of relevant knowledge concerning convective dissolution in CCS projects. Furthermore, understanding the convective dissolution process to predict the long-term field evolution of CO_2_ is important to sequestration technology implementation [8].

During the CO_2_ storage in saline aquifers, the buoyancy of the original formation water is controlled by both thermal (temperature) and compositional (salinity) effects [9]. It is because the solubility of CO_2_ is sensitive to the temperature and salinity conditions of saline aquifers. In those actual ongoing CCS projects, the temperature and salinity conditions vary over a wide range [10]. “Cold” sedimentary basins of cold regions and offshore, with the temperature during brine—CO_2_ interaction below 50 °C are more favorable for CO_2_ storage [11], because CO_2_ attains higher density at shallower depths than in “warm” sedimentary basins, which are characterized by high-temperature gradients where dense-fluid conditions are reached at greater depths. In shallow groundwater systems, the salinity of formation water increases with the formation depth, while the interfacial tension (IFT) increases with the increase in salinity, which is attributed to the hydration of ions [12]. Numerical studies have shown that for the constant pressure and temperature, high-salinity brines decrease the density difference between CO_2_-dissolved brine and original brine, delaying the onset of instability and slowing down the long-term convective mixing [13].

The instability behavior of convective dissolution has been investigated by many researchers. Lindeberg and Wessel-Berg [14] conducted numerical simulation of the stability criteria for the density-driven convection process. It was followed by several other studies that applied linear stability analysis to predict the convection starting time, the dominant wavelength, and wavenumber for the convective fingers [15,16,17].

As the spatial and temporal scales involved make it difficult to analyze these dynamics in situ, the experimental research on convective dissolution at the laboratory scale is receiving more and more attention [18,19,20]. Three main types of experimental methods have been used to analyze CO_2_ convective dissolution in aqueous solutions: PVT (pressure-volume-temperature) reactor experiments [21,22,23], Hele–Shaw cell experiments [7,16,24,25], and bead pack experiments [18,19,26,27]. PVT reactor experimental results can be used to easily quantify the total dissolved CO_2_ mass and mass transfer rate at high pressures and elevated temperatures; however, they have limited ability to visualize the convective dissolution process. In contrast, the Hele–Shaw cell experiment is optimal for visual studies, which are commonly used at ambient conditions to visualize the dissolution process; however, the quantification of the mass transfer rate is not as straightforward as that in PVT reactor experiments. In addition, 3D quantitative measurements can be realized by bead pack experiments, but these experiments must be combined with three-dimensional imaging techniques, such as X-ray computed tomography or a magnetic resonance imaging system. In other words, this method needs equipment with complex instruments, and it is not easy to operate.

Previous experiments in Hele–Shaw cells have stimulated considerable theoretical investigations of convective flows. A Hele–Shaw cell is made of two transparent glass or Plexiglas^®^ plates that are parallel to each other, sealed on the edges, and separated by a narrow gap, which is filled with fluids. Many studies have been conducted with Hele–Shaw cells to reproduce the convective behavior of CO_2_-dissolved in brine with analogue fluids [28,29,30,31]. The most common fluids are propylene glycol (PPG)/water, mixtures of methanol and ethylene glycol (EG-MeOH)/water, and potassium permanganate/water. The main purpose of using analogue fluids is to mimic the density differences of the actual fluids and allow experiments to proceed at normal laboratory conditions, length, and time scales [32]. However, there are some fundamental differences between analogue fluids and CO_2_/brine systems, such as the density-concentration behavior, viscosity differences, and miscible properties [15,33]. These experimental observations of analogue fluids can aid in understanding the dissolution process and provide physical evidence of mixing induced by density-driven convection in CO_2_ geological storage.

Due to the necessity of quantitatively measuring the CO_2_ mass transfer to predict CO_2_ sequestration capabilities, recent visualization experiments have depended on the optical technique applied using different approaches to quantify the mass transfer. Backhaus et al. measured the temporal evolution using an optical shadowgraph and determined the mass transfer rate [31]. Tsai et al. characterized the maximum convective mass flux by measuring the constant speed of the moving interface [34]. Faisal et al. determined the total mass of dissolved CO_2_ by a total carbon analyzer (TC analyzer), which utilizes a catalytic oxidation combustion technique [25]. Rasmusson et al. proposed an experimental method that uses the refractive index of the fluid to quantify the mass flux [35]. However, the previously mentioned methods are either conducted with analogue fluids in CO_2_ convective dissolution experiments or cannot measure the CO_2_ dissolution mass in a timely and successive manner. There is a limited number of quantitative visual experiments in the area of CO_2_ convective dissolution due to the difficulty of measuring the mass transfer quantitatively within the CO_2_/brine system. Furthermore, none of the existing studies have compared the effects of temperature and salinity on convective instability through visualization experiments and simulation.

In this study, we carried out in situ dissolved CO_2_ concentration measurement experiments using a new spectrophotometric method. We also present a numerical analysis of the density-driven convective instability and the dissolved CO_2_ mass in Hele–Shaw cells with respect to different salinity and temperature conditions. The concentration of dissolved CO_2_ was reported, and the convective fingers images during the CO_2_ dissolution process were quantitatively analyzed. The purpose of this study is to provide quantitative visual evidence of saline water convection with dissolved CO_2_ on time and space scales that are easily accessible in the laboratory.

## 2. Experimental

Experiments were carried out under three different temperature and four salinity conditions. For the cases selected here, temperature conditions can be compared to a storage scenario in a “cold” basin of saline aquifers (geothermal gradient equal to 25 °C/km and average surface temperature around 10 °C) and injection into a rather “shallow” reservoir [36]. Furthermore, sodium chloride (NaCl) was selected to represent the salinity in this study, because Na^+^ is one of the most commonly cations that are found in groundwater [37]. The highest salinity condition, 15,000 mg/L, was inspired by data referring to the CO_2_ storage reservoir at Otway Basin [38].

### 2.1. Experimental Setup

**Figure 1:**
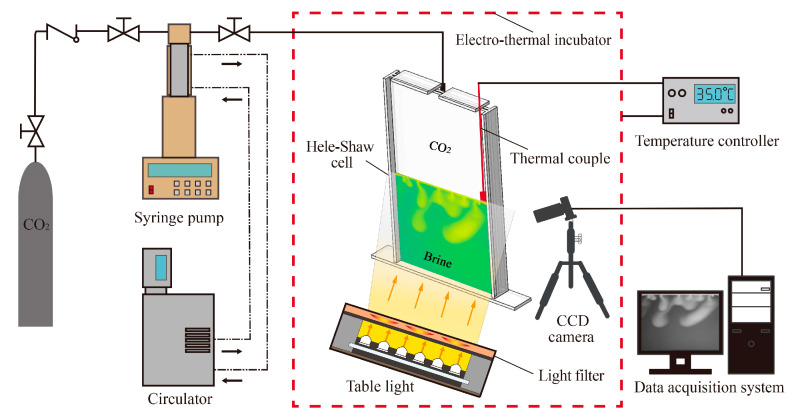
System diagram of the experimental setup.

Figure 1 shows a schematic of the experimental setup in this study. The experimental setup consists of a Hele–Shaw cell, syringe pump, CO_2_ tank, temperature controller, imaging system, and a PC with a data acquisition system. The two main parts of this setup are the temperature control system and imaging system. It should be pointed out that all experiments were performed at atmospheric pressure.

The glass sheets of the Hele–Shaw cell were separated by spacers with a width *b* of 1 mm on all sides except at the top. The vertical and bottom sides of the cell were sealed, and the top of the cell was covered but not completely sealed. The length and height of the internal cell dimensions are 100 and 200 mm, respectively. To conduct this experiment, the bottom space of the cell was filled with brine, and the interface height was 100 mm. CO_2_ gas was introduced from the upper end, forming the stratified Hele–Shaw flows. Under atmospheric conditions, CO_2_ is approximately 1.5 times heavier than air (the molecular weights of CO_2_ and air are 44 g/mol and 29 g/mol, respectively); therefore, the air and the excess CO_2_ can flow outside of the unsealed top.

The main purpose of Hele–Shaw cells is to simulate an environment that is mathematically analogous to 2D flow in porous media. The flow behavior in the Hele–Shaw cell is governed by the same Darcy’s law as flow in porous media. The permeability of the Hele–Shaw cell was calculated using the equation *k* = *d*^2^/12, and the porosity of the cell was considered as 1.

A custom electro-thermal incubator was used to adjust the temperature of the experiments. It was made with thermal insulation material and an aluminum alloy supported frame; a silicone heating plate was attached to the inner surface. The heating plate related to a power source through the conductive electrode, which was connected with a temperature-control adjuster (AT72AAS4R, Autolise Co., Ltd., Dalian, China, 20–80 °C, resolution 0.1 °C). An ungrounded K-type thermo couple was used to monitor the experimental temperature. The temperature control circuit can provide rapid temperature increases and can maintain a constant predetermined temperature. The front side of the incubator was removable, which was convenient for experimental preparation.

The convective fingers images were captured at regular intervals using an MV-EM510 image processing charge-coupled device (CCD) camera (Microvision Digital Image Technology Co., Ltd., Beijing, China.) with a maximum resolution of 2456 × 2058 pixels and an 8-bit output. This high-resolution camera has a 2/3 progressive scan CCD sensor and can capture 15 frames per second. It was directly connected to a computer to facilitate advanced image processing. A CCD camera was positioned before the Hele–Shaw cell, while a monochromatic light filter and a uniform table light were positioned behind the cell. The central wavelength of the filter is 615 nm, which corresponds to the maximum wavelength of the pH indicator. The CCD relative spectral response near this wavelength is up to 80%, which meets the experimental requirements. Due to the excellent modulation characteristics of the table light, the infused current of the light source was modulated with a light controller to make the incident light brightness change with a signal from the modulator. The light controller is equipped with an RS-232 interface, which can be used for reading and setting light source parameters on the controller panel. The CCD camera, Hele–Shaw cell, light filter, and table light were placed in a row at a proper distance and height so that the camera could focus on the target area. These positions remained the same for subsequent experiments. To avoid external reflections, the above four devices were placed in the custom incubator as a darkroom where the uniform table light was the only source of light.

The pump used for CO_2_ injection is an ISCO pump (260D, Teledyne ISCO, Lincoln, NE, USA), which is a precise and accurate laboratory pump with a minimum injection rate of 0.001 mL/min, maximum injection rate of 107 mL/min, and accuracy of 0.5%. This ISCO pump was connected to the CO_2_ tank via a tube and a pressure regulator. Before the experiment, it was filled with CO_2_ and maintained at atmospheric pressure. The temperature control jacket encircles the cylinder of the pump, allowing water to circulate through the jacket to maintain the temperature of CO_2_ within the cylinder.

### 2.2. Material and Properties

A pH indicator, bromocresol green, was added to the brine for visualizing the dissolved CO_2_ convective fingers. Under our experimental conditions, dissolved CO_2_ into brine at atmospheric pressure and the solution pH value decreased to approximately 4. The transition range of bromocresol green is 3.8–5.4; therefore, the pH range of the CO_2_-dissolved brine was within the pH range of the indicator. To match the intensity of the incident light and obtain the highest quality images, several trial and error experiments were performed to determine the bromocresol green concentration. For our experimental equipment, the optimum concentration of bromocresol green is 2.5 × 10^−4^ mol/L. The small amount of bromocresol green has no effect on the properties of the water.

The working solutions were prepared by successive dilution. The pH indicator solution was prepared by dissolving 0.014 g of bromocresol green in 2 mL of 0.025 mol/L sodium hydroxide and adding de-ionized water to obtain a total volume of 200 mL. Then, sodium chloride (NaCl) was dissolved in the pH indicator solution to obtain different concentrations of brine. CO_2_ with 99.99% purity was used for experiments.

The density of aqueous CO_2_ solutions was calculated according to Duan [39]. The viscosities of the brine solutions were determined by using a rotational viscometer (NDJ-5S Jingtian Co., Ltd., Shanghai, China). The diffusion coefficient was calculated by the Unver and Himmelblau [40] correlation, which related the diffusion coefficients to temperature only. Table 1 presents the thermodynamic properties of the fluids used in the experiments.

### 2.3. Experimental Procedure

The following procedure was used to prepare and observe the density-driven convection of dissolved CO_2_ into brine. (1) Prior to each experiment, the Hele–Shaw cell was thoroughly cleaned, and the pH indicator solution and brine were prepared. (2) The cell was partially filled to a specified height (10 cm) with brine containing the pH indicator. Next, the CCD camera, Hele–Shaw cell, light filter, and table light were put into a specific position within the electro-thermal incubator. (3) The Hele–Shaw cells were connected to the ISCO pump via a tube, and a specific incident luminous intensity was obtained by opening and adjusting the infused current of the light. (4) The electro-thermal incubator was closed, and the electro-thermal incubator and water bath were set to the desired temperature and left for approximately half an hour to ensure that the temperature had stabilized. (5) The CCD camera was turned on to capture the dynamics at successive times (0.1 fps), and the valve of the pump was opened to introduce CO_2_ into the cell at a constant flow rate (0.5 mL/min). (6) The experiments were repeated three times and continued until the gray value of the images remained constant, which indicated that mixing was complete and the system had equilibrated.

## 3. Theory and Methods

### 3.1. Dimensionless Parameters

To better understand the density-driven convection due to CO_2_ dissolution, some dimensionless parameters were used to describe and compare the experimental results for different scales and conditions.

The Rayleigh number is the main dimensionless number to describe the gravitational instabilities; it is the ratio between the buoyancy and diffusion forces and was defined as follows:

(1) $$\mathrm{Ra}\;=\;\frac{\mathrm{kgh}\Delta \rho }{\phi \mu D}$$

where *k* is the permeability of the porous medium, Δρ refers to the maximum density difference between the two miscible fluids, *h* is the characteristic length and equals the height of the interface, *μ* is the viscosity of the solution, *D* is the diffusion coefficient, and 𝜙 is the porosity.

The dimensionless flux, characterized by the Sherwood number, *Sh*, is the ratio of the convection mass flux to the diffusive flux

(2) $$\mathrm{Sh}\;=\;\frac{F_{c}}{\phi \Delta cD/h}$$

where the convection flux $F_{c}=\phi u\Delta c$ is based on the concentration difference between the liquids, Δc is the concentration difference, and u is the characteristic velocity.

### 3.2. Spectrophotometric Method

Interventionary studies involving animals or humans, and other studies that require ethical approval, must list the authority that provided approval and the corresponding ethical approval code.

In this study, we carried out in situ dissolved CO_2_ concentration measurement experiments using a spectrophotometric method. According to the Beer–Lambert law [41], light attenuation is related to the properties of the material through which it travels. CO_2_ dissolution into saline water was observed under transmitted monochromatic light, and a CCD camera was used to capture images. The gray value of these images are processed using the open-source software Image *J* [42]; each image contains gray values between 0 (dark) and 255 (bright) and was analyzed to quantify the CO_2_ convective dissolution over time.

When CO_2_ dissolves in saline water, it reacts with H_2_O to form carbonic acid (H_2_CO_3_), which dissociates into bicarbonate ${\mathrm{HCO}}_{3}^{-}$ and carbonate ${\mathrm{CO}}_{3}^{2-}$ ions instantaneously, according to the following equilibrium equations:

(3) $${\mathrm{CO}}_{2}\left(g\right)\;⇌{\;\mathrm{CO}}_{2}\left(\mathrm{aq}\right)$$

(4) $${\mathrm{CO}}_{2}\left(\mathrm{aq}\right){\;+\;H}_{2}O\;⇌{\;H}^{+}{+\;\mathrm{HCO}}_{3}^{-}$$

(5) $${\mathrm{HCO}}_{3}^{-}\;⇌{\;H}^{+}{+\;\mathrm{CO}}_{3}^{2-}.$$

The equilibrium constant for Equation (4) is several orders of magnitude greater than that for Equation (5). For the convenience of calculation, Equation (4) is neglected. To determine the dissolved CO_2_ concentration, a suitable mathematical analysis is used.

Absorbance (*A*) is the natural logarithm of the ratio of incident to transmitted radiant power through a material, and the absorbance *A* can be calculated as follows:

(6) $$A\;=\;\mathrm{lg}(\frac{I_{v0}}{I_{v}})$$

where $I_{v0}$ is the luminous intensity passing through the empty Hele–Shaw cell, and $I_{v}$ is the luminous intensity after passing through the Hele–Shaw cell with solution.

As mentioned earlier, the driving circuitry of the table light is equipped with light brightness feedback to control the output light brightness. The light controller provides an adjustment function for 256 luminance levels. We measured the grayscale G in the region of interest (ROI) under several different luminance levels $L_{l}$, concluded that the grayscale is linearly dependent on the luminance within a certain luminance range, and the relationship expressed as follows:

(7) $$G\;=1.2L_{l}+45,\;L_{l}\;\in \;[\;28,\;175\;].$$

The maximum luminance level within the linear correlation interval $L_{l}$ = 175 was selected as the incident light source luminance. In addition, luminance is a photometric measure of the luminous intensity per unit area of light traveling in a given direction, which is normally obtained by dividing the luminous intensity by the light source area.

Combining Equations (6) and (7), and for convenience in absorbance calculation, the ratio of luminous intensity can replace by the ratio of luminance level. The absorbance *A* can be translated as follows:

(8) $$A=1g\left(\frac{L_{l_{0}}}{L_{l}}\right)=-1g\left(\frac{G\;-\;45}{G_{0}\;-\;45}\right)$$

where $G_{0}$ is the gray value in the ROI of the empty Hele–Shaw cell image, and *G* is the gray value in the ROI of the Hele–Shaw cell with solution.

As the Beer–Lambert law [41] points out, absorbance is correlated with the concentrations of attenuating species as well as the thickness of the solution:

(9) A = α·b·c

where *α* is the absorbance coefficient, *b* is the thickness of the solution, and *c* is the concentration of the absorbent solute.

The pH indicator consists of organic molecules that exhibit acid–base properties. The acidic form of the indicator (HIn) has a different color compared to the conjugate base (In^−^). A general equilibrium expression for the indicator is as follows:

(10) $$\mathrm{HIn}\;⇌{\;H}^{+}{+\;\mathrm{In}}^{-}.$$

The equilibrium constant of Equation (10) is as follows:

(11) $$K_{\mathrm{HIn}}{\;=\;[H}^{+}]\;\times \;\frac{{[\mathrm{In}}^{-}]}{\left[\mathrm{HIn}\right]}$$

where [ ] indicates the molar concentration of the species inside the square brackets.

The logarithm of Equation (11) can be simplified to the following:

(12) $${\mathrm{lg}(K}_{\mathrm{HIn}})\;=\;\mathrm{lg}\left(\frac{{[\mathrm{In}}^{-}]}{\left[\mathrm{HIn}\right]}\right)-\mathrm{pH}.$$

The hydrogen ion (H^+^) released due to the CO_2_ dissolution changes the ratio of [HIn] to [${\mathrm{In}}^{-}$], which causes a color variation in the solution. The absorbance coefficient of ${\mathrm{In}}^{-}$ is $\alpha_{-}$, and the absorbance coefficient of HIn is *α*_0_. The absorbance of the solution is as follows:

(13) $$A\;=\;\alpha_{-}\cdot b\cdot \left[{\mathrm{In}}^{-}\right]+\alpha_{0}\cdot b\cdot \left[\mathrm{HIn}\right].$$

The pH indicator concentration *c* is equal to:

(14) $$c\;=\;\left[{\mathrm{In}}^{-}\right]\;+\;[\mathrm{HIn}].$$

The absorbance of the indicator in a strong acid or strong alkali environment is as follows:

(15) $$A_{\left(\mathrm{HIn}\right)}{\;=\;\alpha }_{0}\cdot b\cdot \left[\mathrm{HIn}\right]{\;=\;\alpha }_{0}\cdot b\cdot c$$

(16) $$A_{{(\mathrm{In}}^{-})}\;=\;\alpha_{-}\cdot b\cdot \left[{\mathrm{In}}^{-}\right]\;=\alpha_{-}\cdot b\cdot c.$$

By using the extreme value of absorbance in Equation (13) and combining Equations (10), (11) and (14), the following equations are derived:

(17) $$A\;=\;\frac{A_{{(\mathrm{In}}^{-})}}{c}{[\mathrm{In}}^{-}]\;+\;\frac{A_{\left(\mathrm{HIn}\right)}}{c}\left[\mathrm{HIn}\right]$$

(18) $$\left[{\mathrm{In}}^{-}\right]\cdot \left(A_{\left({\mathrm{In}}^{-}\right)}-A\right)\;=\;[\mathrm{HIn}]\cdot (A-A_{\left(\mathrm{HIn}\right)})$$

(19) $$\mathrm{lg}(\frac{K_{\mathrm{HIn}}}{{[H}^{+}]})\;=\mathrm{lg}(\frac{{[\mathrm{In}}^{-}]}{\left[\mathrm{HIn}\right]})=\mathrm{lg}\left(\frac{A-A_{\left(\mathrm{HIn}\right)}}{A_{\left({\mathrm{In}}^{-}\right)}-A}\right)$$

(20) $$\mathrm{lg}\left(\frac{A-A_{\left(\mathrm{HIn}\right)}}{A_{\left({\mathrm{In}}^{-}\right)}-A}\right)\;=\;\mathrm{pH}\;-pK_{HIn}$$

where $\mathrm{lg}\left(\frac{A-A_{\left(\mathrm{HIn}\right)}}{A_{\left({\mathrm{In}}^{-}\right)}-A}\right)$ is a factor related to absorbance and is proportional to the solution pH. A series of tests were conducted to calibrate the linear relationship between pH and $\mathrm{lg}\left(\frac{A-A_{\left(\mathrm{HIn}\right)}}{A_{\left({\mathrm{In}}^{-}\right)}-A}\right)$. Hydrochloric acid (HCl) or sodium hydroxide (NaOH) was added to the pH indicator solution to obtain five solutions with different pH values that were within the functional range of the indicator. The pH value was measured using a pH meter.

The extreme values of absorbance, *A*(HIn) and *A*(${\mathrm{In}}^{-}$), as shown in Table 2, were derived from the gray value of the images for the pH = 1 and pH = 10 indicator solutions by titration with HCl or NaOH.

**Figure 2:**
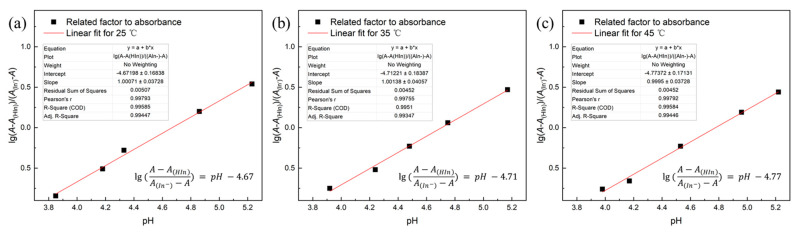
Linear fitting between log((*A-A*_HIn_)/(*A*_In-_*-A*)) and pH at different temperatures. Each point represents the average of three experiments, (**a**) 25 °C, (**b**) 35 °C, (**c**) 45 °C.

As shown in Figure 2, there is good linearity between $\mathrm{lg}\left(\frac{A-A_{\left(\mathrm{HIn}\right)}}{A_{\left({\mathrm{In}}^{-}\right)}-A}\right)$ (the factor related to absorbance) and solution pH. This curve is the standard curve for the dissolved CO_2_ concentration measurement experiments, which is used to calculate [H^+^] from the absorbance.

Based on the requirement for electrical neutrality in the solution, the following charge balance equation is obtained:

(21) $$\left[{\mathrm{HCO}}_{3}^{-}\right]\;+\;\left[{\mathrm{In}}^{-}\right]{\;=\;[H}^{+}]+\;\left[{\mathrm{Na}}^{+}\right]$$

where the $\left[{\mathrm{Na}}^{+}\right]$ is equal to the concentration of sodium hydroxide in the pH indicator solution.

Using the equilibrium constant *K*_HIn_ (Equation (11)) and the indicator concentration *c* = 2.5 × 10^−4^ mol/L from Equation (18), [In^-^] can be written as follows:

(22) $$\left[{\mathrm{In}}^{-}\right]\;=\;\frac{c}{10^{-\mathrm{pH}}\cdot {\;K}_{\mathrm{HIn}}^{-1}\;+\;1}.$$

By combining the standard curve with Equations (17)–(19), ${[\mathrm{HCO}}_{3}^{-}]$ can be calculated.

The equilibrium expression involving CO_2_ (aq) (Equation (5)) at different temperatures [43] can be combined with the equilibrium constant for the reaction and written as follows:

(23) $$\left[{\mathrm{CO}}_{2}\left(\mathrm{aq}\right)\right]\;=\;\frac{\left[H^{+}\right]\;\cdot {\;[\mathrm{HCO}}_{3}^{-}]}{K_{{\mathrm{CO}}_{2}\left(\mathrm{aq}\right)}}.$$

Finally, the concentration of dissolved CO_2_ consists of two parts: [CO_2_(aq)] and [${\mathrm{HCO}}_{3}^{-}$]:

(24) $${[\mathrm{CO}}_{2}{(\mathrm{dissolved})]\;=\;[\mathrm{CO}}_{2}{(\mathrm{aq})]\;+\;[\mathrm{HCO}}_{3}^{-}].$$

### 3.3. Numerical Simulation

The numerical model used in our simulation was built similar to the experimental configuration considering the CO_2_-saturated water interface in the Hele–Shaw cell. The top boundary is at atmospheric pressure conditions. Bottom and sides boundaries are considered as no-flow boundaries. Simulation was performed using STOMP (Subsurface Transport Over Multiple Phases), and the operational mode is STOMP-CO2.

The governing equations of flow and concentration field for such a system are as follows:

(25) $$\frac{\partial }{\partial t}\left[\sum^{\;}\left(\rho \phi S\omega^{i}\right)\right]=-\sum^{\;}\nabla \left(\rho \omega^{i}V\right)-\sum^{\;}\nabla \left(F^{i}\right)+\sum^{\;}\left(\omega m_{d}\right)$$

where *S* is the saturation, *ω* is the mass fraction, *i* represents the species (H_2_O or CO_2_), and *m_d_* is the mass rate density.

Darcy’s law is used to compute the advective fluxes ***V***:

(26) $$V=-\frac{k_{r}k}{\mu }\left(\nabla P+\rho gh\right).$$

The diffusive flux ***F*** is calculated considering molecular diffusion and neglect dispersion:

(27) $$F^{i}=-\;\rho \phi S\frac{M^{i}}{M}\left(\tau D^{i}\right)\nabla \chi^{i}$$

where *M* is molecular weight, τ is tortuosity, and χ is mole fraction.

The saturation–capillary pressure function is that of van Genuchten [44]:

(28) $$\bar{S_{el}}={\left[1+{\left(\alpha h\right)}^{n}\right]}^{-m};\;m=1-\frac{1}{n};\;\bar{S_{el}}=\frac{S_{l}-S_{r}}{1-S_{r}}.$$

The relative permeability function of van Genuchten (α = 500; *n* = 5) is associated with the Mualem porosity distribution model [45,46]:

(29) $$k_{r}=\sqrt{\bar{S_{el}}}\left[{\left(1-{\left(1-{\left(\bar{S_{el}}\right)}^{1/m}\right)}^{m}\right)}^{2}\right].$$

A detailed description of all the state equations used in STOMP-CO2 can be found in White et al. [47]. The mesh sensitivity analysis was conducted, and we concluded that the uniform mesh of 100 × 100 (grid cell length and height equal to 1 mm) was adequate when the CO_2_ mass transfer remained stable. The simulation was perturbed with non-regular sinusoidal perturbation introduced on the initial concentration profile just below the interface.

## 4. Results and Discussion

The main aim of this work was to establish a new spectrophotometric method for the quantitative visualization of CO_2_ convective dissolution in brine, investigate the effects of salinity and temperature on convective instability and the mass of CO_2_ dissolved, and finally use the experimental data for simulation validation.

### 4.1. Development Regimes and Morphology of Instabilities

**Figure 3:**
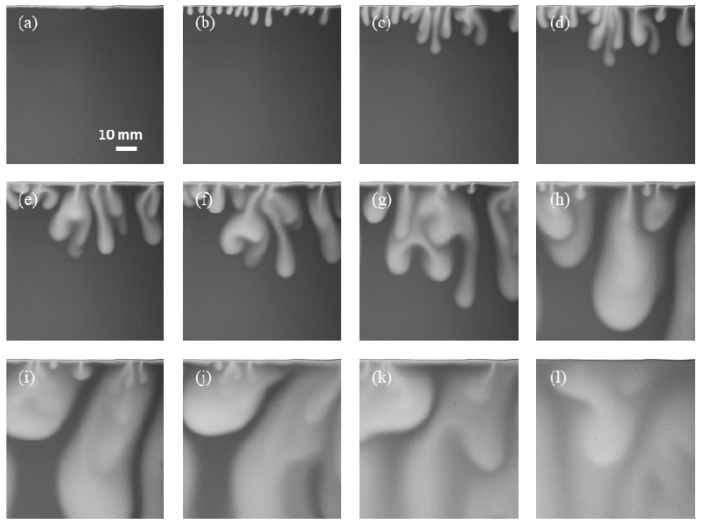
CO_2_ dissolution images for Exp. No. 1. (**a**) 100 s, (**b**) 300 s, (**c**) 600 s, (**d**) 800 s, (**e**) 1200 s, (**f**) 1600 s, (**g**) 2500 s, (**h**) 5000 s, (**i**) 7000 s, (**j**) 9000 s, (**k**) 11,000 s, and (**l**) 18,000 s.

Figure 3 shows successive images of the CO_2_ dissolution in deionized water at 25 °C. The selected ROI has a dimension of 75 mm × 75 mm. When CO_2_ was injected into the Hele–Shaw cell with brine, CO_2_ started to dissolve into the brine, and the CO_2_-dissolved brine was denser than the native brine. The CO_2_-dissolved brine, which is shown as a thin and bright line layer on the images (Figure 3a), appeared almost instantaneously behind the interface once the CO_2_ was injected into the Hele–Shaw cell, and this indicated the beginning of the reaction. At the initial stage, the only active mechanism is diffusion, and the denser brine layer below the interface is relatively stable; this stage refers to the diffusion dissolution regime. Next, the CO_2_ mass influx produced an increase in density, the density difference caused instability, small fingers formed, and eventually, convection was triggered.

At early times, convective fingers grow independently (Figure 3b). As time passes, there are interactions between adjacent fingers. The small fingers first merged at the interface, and then the tips of the fingers tended to coalesce into longer and larger fingers with a few seconds of elapsed time. The wave numbers were determined by counting the number of fingers at the top (interface) and the bottom (tip of fingers) using the length scale. It is clearly seen from 600 to 11,000 s (Figure 3c–k) that as the process proceeds, convective fingers are fully developed, and wave numbers decreased both at the top and bottom. The CO_2_-dissolved brine moved downwards, and the native brine moved upwards to the interface. Meanwhile, new fingers emerged from the interface between existing fingers and follow the same behavior. Several dynamic regimes of CO_2_ convective dissolution have been proposed in the literature [37,48]; this period in our experiments is known as the convective regime. Eventually, the downward fingers touched the bottom boundary and became blurred, and this resulted in the decay of the dissolution as the system entered the shutdown regime (Figure 3k–l).

When the convection regime is active, the dissolution rate of CO_2_ into the brine is greater. As can be seen from Figure 3, within 100 s after introducing CO_2_ into the top of the brine, only a thin-layer area below the interface became bright, and diffusion dominates the mass transport. However, from time *t* = 600 s, when convection dominates the mass transport, a larger area of brine became bright, which shows the direct effects of convection on the enhanced CO_2_ dissolution. The development regimes variation of density-driven convective are comparable to the reported results [21]. We will perform a quantitative analysis of CO_2_ dissolution in the later section.

### 4.2. Effects of Brine Salinity and Temperature

**Figure 4:**
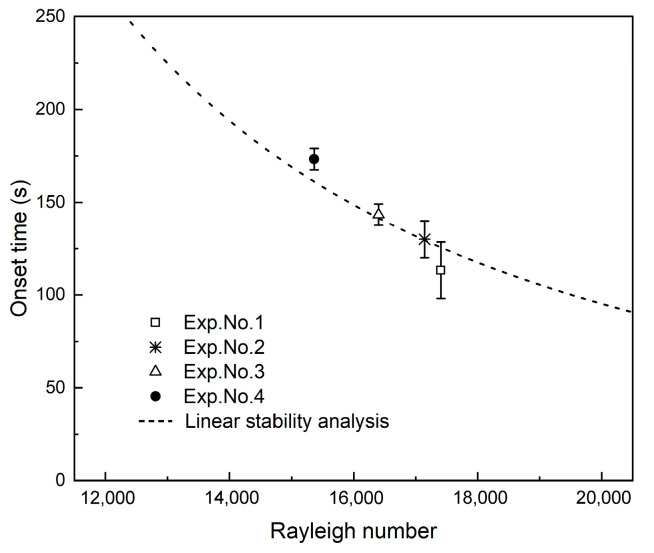
Approximate experimental measurement and linear stability analysis of convection starting time changes with Rayleigh number for Experiments (Exps.) No. 1–4.

To investigate the effects of salinity on convective instability of CO_2_ dissolution, we analyzed the convection starting time and the length of fingers. The convection starting time is the time at which the convective fingers can be observed. With the careful observation of our experimental images, the approximate convection starting time (based on the first appearance of fingers at the interface) is recorded. Under the experimental conditions in this study, the convection starting time occurred within 300 s. Analytical and numerical calculations indicated that the convection starting time shows a relation with $\approx 1/Ra^{2}$ [49]. We plotted the average value of the convection starting time change with Rayleigh number, as shown in Figure 4. The same behavior is observed in the variation of the convection starting time in the different salinities by the experimental measurement and linear stability analysis calculation. The higher the salinity, the smaller the *Ra* and the longer the convection starting time. In conclusion, increasing salinity changes the convection starting time in an undesirable way.

**Figure 5:**
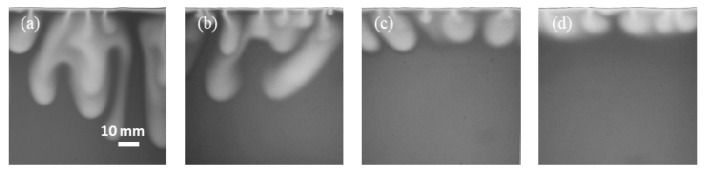
CO_2_ dissolution images at 3000 s for different salinities of the brine: (**a**) Exp. No. 1; (**b**) Exp. No. 2; (**c**) Exp. No. 3; (**d**) Exp. No. 4.

**Figure 6:**
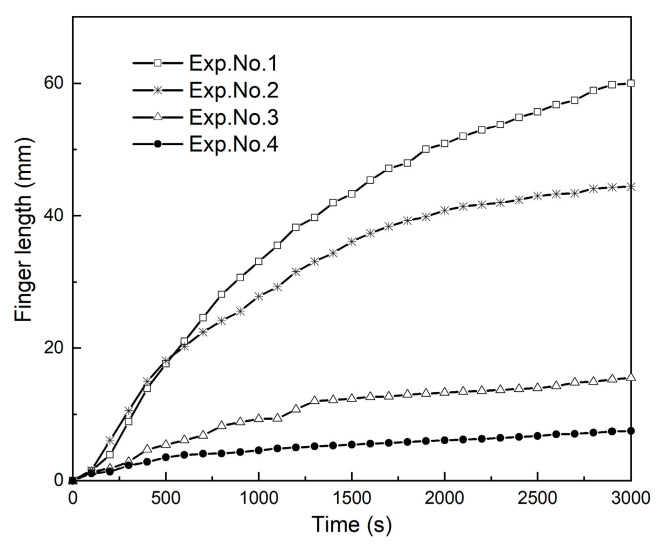
Temporal evolution of the finger’s length at 25 °C for Exps. No. 1–4.

Figure 5 shows the CO_2_ convective dissolution images at 3000 s for different salinities. The length of the fingers was measured from the interface to the tip of the fingers. These four experiments exhibit different instability behavior in different fingering pattern. Lower salinity results in a faster development of instabilities such that the growth rate of fingers is significantly stronger. The temporal evolution of the fingers length in different salinity conditions is shown in Figure 6, where the observed growth is not linear. The change in fingers length at the initial times is fast compared with final times. After the short diffusion stage, a sharp increase indicates the evolution of a convective process.

When CO_2_ fingers occurred at the highest salinity (Exp. No. 4), compared with other conditions, it was the shortest growing fingers. The variation between the three curves in Figure 6 is due to the differences in density caused by salinity. The CO_2_ solubility in brine decreased with increasing salinity, and the density difference between brine with and without dissolved CO_2_ is decreased [50]. The viscosity of aqueous solutions also increased with increasing salinity because it is based on the density variation. Dissolved ionic compounds increase the density and therefore its viscosity [21]. The density difference is the major factor that determines how fast the convective fingers move [51]. In short, salinity has a negative effect on CO_2_ convective dissolution. With increasing salinity, the length of fingers decreases, and the fingers growth rate decreases.

**Figure 7:**
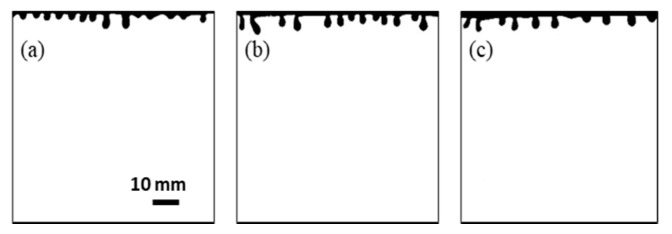
Binary images of the largest wave number appearance for different temperatures: (**a**) Exp. No. 1, *t* = 140 s; (**b**) Exp. No. 5, *t* = 230 s; (**c**) Exp. No. 6, *t* = 270 s.

Our experiments were also performed under different temperature conditions, varying the temperature from 25 to 45 °C. The increase in temperature led to the increased Rayleigh number, and the appearance time of the largest wave number is delayed, as shown in Figure 7. The CO_2_-affected area of the convective pattern was analyzed for different temperature conditions. This area corresponds to the ratio of the fingers area to the total aqueous phase area and could be interpreted as an approximate measure of CO_2_ dissolution. The larger the affected area, the larger the amount of dissolved CO_2_. To distinguish the CO_2_-affected area from the area without dissolved CO_2_, the images were converted into binary images through threshold segmentation, and the affected area can be clearly shown on the background.

**Figure 8:**
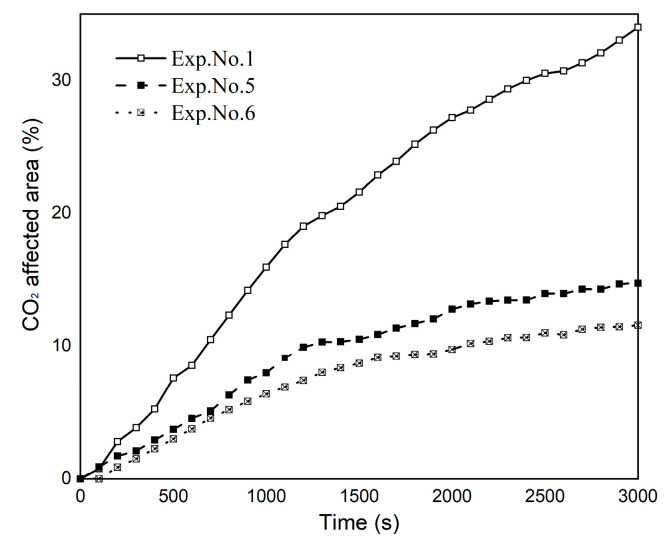
Temporal evolution of the CO_2_-affected area at 25 °C, 35 °C, and 45 °C for Exps. No. 1, 5, and 6.

To make a qualitative comparison, the Image*J* area fraction measurement function was applied to obtain the percentage of the CO_2_-affected area. The affected areas over different temperature experiments are plotted as a function of time in Figure 8. With CO_2_ dissolution, the affected area rapidly increased and then gradually stabilized. It should be noted that due to the temperature difference between these experiments, the CO_2_-affected area in deionized water at 25 °C was larger than the others, and the final values of the CO_2_-affected area decreased with increasing temperature.

Based on thermodynamic theory, the apparent molar volume, that is one parameter of the CO_2_ aqueous solution density, is a function of temperature [52,53]. The increase in the experimental temperature leads to a decrease in the CO_2_ aqueous solution density, which further causes the density difference to decrease. The density difference in the experimental fluids is detailed in Table 1. The density difference is the driving force for convection, and the density difference caused by dissolved CO_2_ increases the CO_2_-affected area. In addition, as CO_2_ reacts with water, its solubility depends on the temperature condition, and CO_2_ solubility decreases with temperature under isobaric conditions [54]. The lower the solubility, the less amount of CO_2_ that could react with water. From the curves, it can be concluded that with increasing temperature, the CO_2_-affected area decreases, and the amount of dissolved CO_2_ decreases.

### 4.3. Determination of the Mass of Dissolved CO_2_

To determine and compare the concentration of dissolved CO_2_ under all experimental conditions, we selected the first 15,000 s (250 min) of the experiment as the characteristic time. Before running the actual experiments, reliability tests were performed using HCl and NaOH solutions of known pH values to verify the accuracy of the method. For three different pH values, the experimental error was found to be less than 5%. In addition, we also measured the solution absorbance with different indicator concentrations and calculated the mass absorption coefficient of the pH indicator at the maximum wavelength. The experimental value has a small error (3.8%) to the property parameter. It has also been suggested that the experimental system is reliable for measuring absorbance.

**Figure 9:**
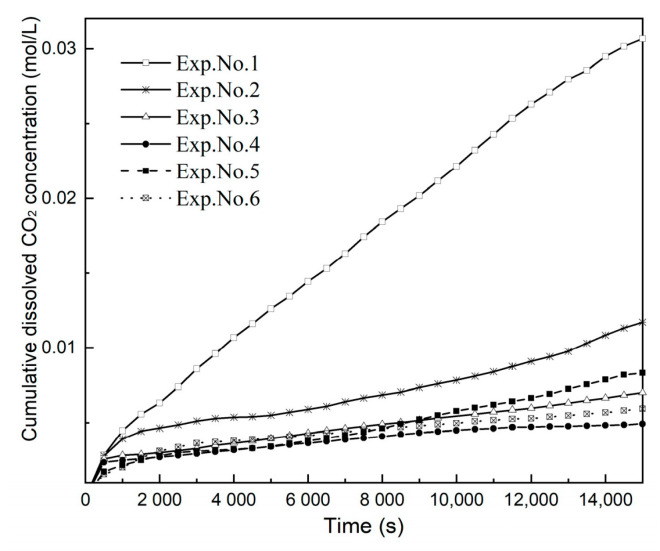
Cumulative dissolved CO_2_ concentration profiles as a function of time.

As described in Section 3.2, the cumulative dissolved CO_2_ concentration was determined by the gray value of the images combined with spectrophotometric analysis. Figure 9 shows the change in the cumulative dissolved CO_2_ concentration for different experimental temperatures and salinities. The dissolved CO_2_ concentration in Exp. No. 1 is greater than all other curves, and this is due to the decrease in salinity and temperature.

**Figure 10:**
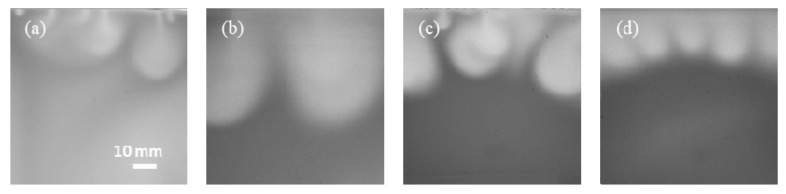
CO_2_ dissolution images at 15,000 s for different conditions: (**a**) Exp. No. 1; (**b**) Exp. No. 2; (**c**) Exp. No. 3; (**d**) Exp. No. 4.

The solubility of Experiment (Exp.) No. 1 at 15,000 s is 1.34 kg/m^3^, and the errors between our experimental result and those of the solubility model of Duan et al. [39] is 6.3%. However, under other experimental conditions, the solubility of our experiments at 15,000 s deviates from the model because the calculated data for convection are not fully developed (see Figure 10); as the bright area of the images increases, the amount of dissolved CO_2_ becomes larger.

After calculating the cumulative concentration of dissolved CO_2_, the cumulative mass of dissolved CO_2_ is calculated. Fick’s second law and the following equation, presented by Amir Taheri et al. [55], are used to calculate the dissolved CO_2_ mass of diffusion dissolution:

(30) $$M\left(t\right)=2C_{0}\sqrt{\frac{Dt}{\pi }}.$$

In the experimental setup suggested here, the concentration is maintained at a constant *C*_0_. Here, the CO_2_ equilibrium concentration at the interface was determined by the solubility model [39].

**Figure 11:**
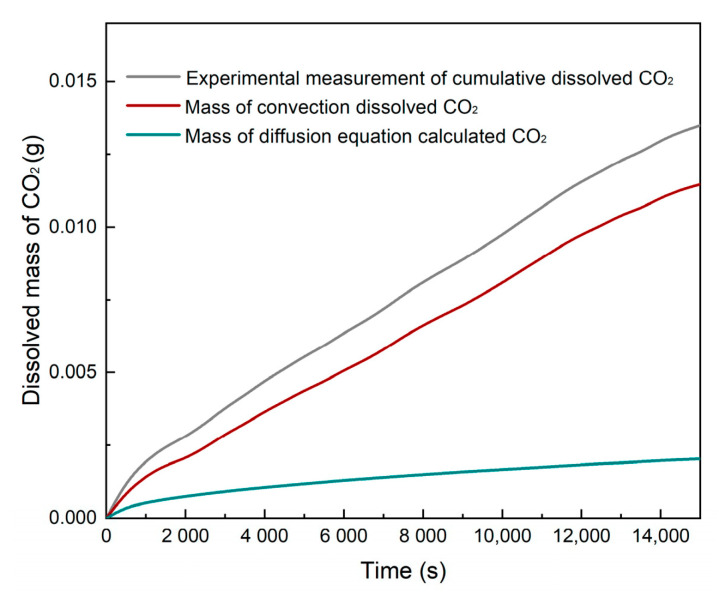
Experimental measurement of dissolved CO_2_, convection of dissolved CO_2_, and calculated mass of CO_2_ from the diffusion equation during Exp. No. 1 within 15,000 s.

**Figure 12:**
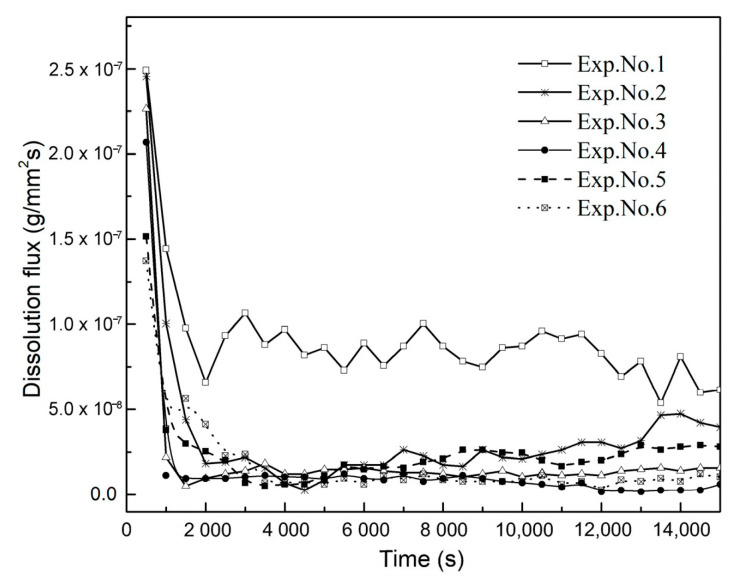
Changes in CO_2_ dissolution flux within 15,000 s.

Reducing the diffusion dissolution of CO_2_ from cumulative dissolved CO_2_ results in the convective dissolution of CO_2_. Figure 11 displays the dissolved mass of CO_2_ of Exp. No. 1 for different dissolution mechanisms. By comparing the mass of diffusion dissolved CO_2_ to convection dissolved CO_2_, the crucial role of convection on the enhancement of CO_2_ mass transfer can be seen. The curve also indicates that the convective dissolution effect increases within 15,000 s. By differentiating from the mass of cumulative dissolved CO_2_, the dissolved CO_2_ flux for each experiment is reached. This investigation is presented in Figure 12.

The CO_2_ dissolution flux curves have different maximum values at the initial convection regime, and they decrease while increases in temperature and salinity cause a decrease in the dissolution flux. Earlier studies on convective systems have focused on the Sherwood number and Rayleigh number. Under our experimental conditions and by increasing salinity and temperature, variables such as density differences and solubility would decrease, the viscosity and diffusion coefficient would increase; thus, their total effects reduce the Rayleigh number.

**Figure 13:**
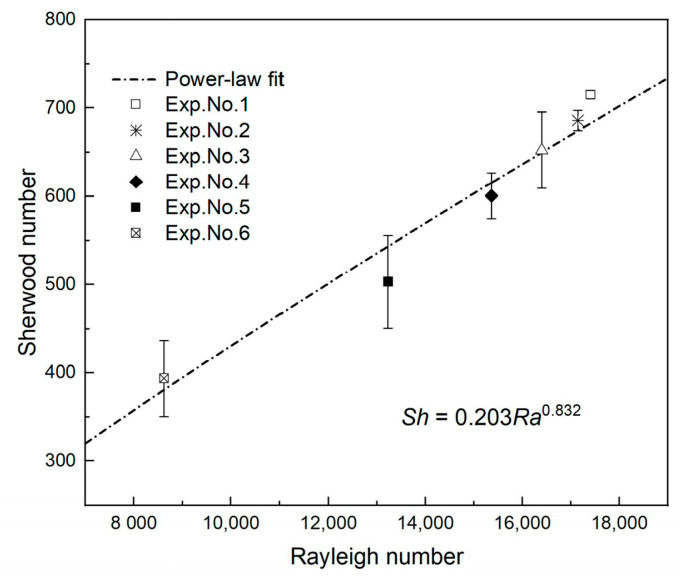
Sherwood number as a function of Rayleigh number for the different experiments.

To find a relationship between *Ra* and *Sh*, the maximum dissolution flux was used. By plotting *Sh* against *Ra*, a relationship is obtained as shown in Figure 13. Here, *Sh* scales with *Ra* as $Sh=0.203Ra^{0.832}$. This agrees with existing numerical [28,56] and experimental [21,31] results with Rayleigh number exponents approximately equal to 4/5. These slight variations in the value of the exponent are attributed to the error in calculating the CO_2_ dissolution flux, the differences in fluid systems and the experimental setups. Figure 13 displays that *Sh* increases with increasing *Ra*, which means that the mass transfer flux of CO_2_ dissolution increases with salinity and as the temperature decreases. If the physical parameters of the candidate field are known, the dimensionless CO_2_ dissolution flux can be estimated, thereby helping to examine the strength of geological CO_2_ storage.

### 4.4. Comparison between Experimental and Simulation Results

**Figure 14:**
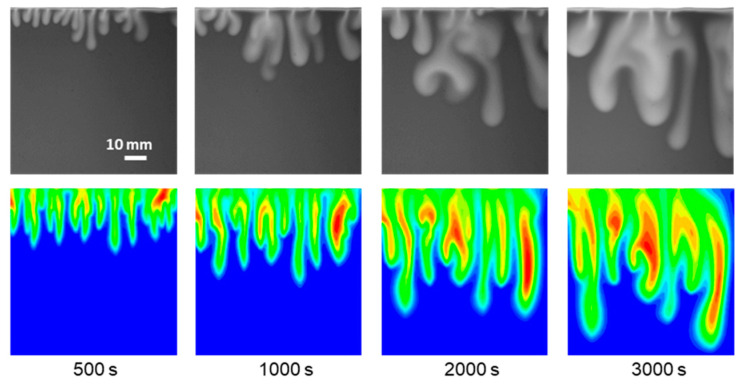
Comparison of convective fingers in experimental and simulation for the CO_2_ dissolution in deionized water at 25 °C.

The numerical simulation uses the experimental results for comparison and validation of the patterns of convective fingers and the mass of dissolved CO_2_. Figure 14 shows the morphology of the fingers in experiment and simulation for the CO_2_ dissolution in deionized water at 25 °C. The simulation results and experimental results accord with very well considering the fingers pattern at the same times, and the fingers become longer and thicker over time. Compared with the experimental results, the simulation show that the rate of fingers growth is slightly faster. It is because the boundary condition of constant saturation in dissolved CO_2_ at the top of the Hele–Shaw cell shorten the time for CO_2_ to diffuse into the aqueous phase.

**Figure 15:**
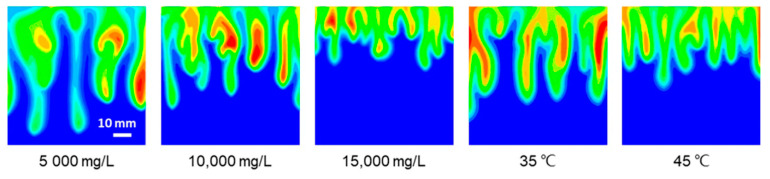
Simulation result of CO_2_ aqueous mass fraction profiles under the corresponding condition of Exp. No. 2–6 at 3000 s.

Figure 15 simulates Experiments 2–6 of different salinity and temperature conditions at 3000 s. The variation trend of fingers length and influence area in the simulation is similar to the experimental results. The increase in brine salinity and temperature adversely affects the growth and development of the fingers. Moreover, the average position of the fingertip in the simulation is lower in comparison to the experiments (see Figure 5).

**Figure 16:**
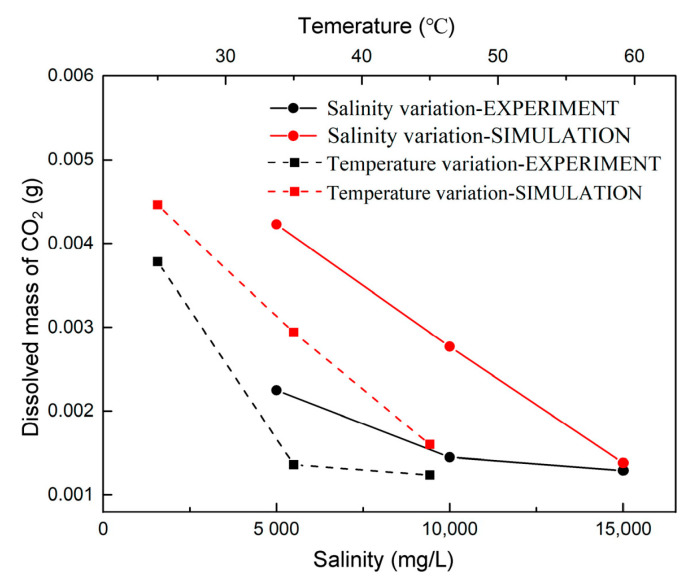
Comparison of cumulative dissolved CO_2_ in the experimental and simulation at 3000 s.

Figure 16 compares the total mass of dissolved CO_2_ at 3000 s for different conditions. The order of magnitudes for the dissolved CO_2_ mass in the simulation is the same as that in the experiments. However, there are some differences between the simulation and experimental results for those salinity and temperature variations cases. The numerical simulations overestimated the dissolved CO_2_ mass affected by salinity and temperature. Comparison of the experimental results with the numerical results shows that the dissolved CO_2_ mass decreases with the increase of salinity and temperature. Moreover, the regularity of the dissolved CO_2_ mass in the simulation (red lines) is more than the experiments (black lines). The assumptions used for the simulation interface seemed to be the primary cause for the discrepancy. The main similarity between experimental and simulation results is that CO_2_ convective dissolution is more effective for CO_2_ storage reservoir with lower salinity and temperature.

## 5. Conclusions

This study developed a quantitative method for analyzing the density-driven convective instability and measuring the mass transfer of CO_2_ dissolution. Several experiments and simulation were performed to investigate the effects of brine salinity and temperature on CO_2_ dissolution relevant to CO_2_ storage in saline aquifers. Based on the analysis of the results, the following conclusions can be drawn:

1. There are three different regimes of instability development: diffusion dissolution regime, convection diffusion regime, and shutdown regime. The course and development of the convective fingers go through generation, propagation, coalescence, and re-initiation.
2. Brine salinity has a negative effect on the CO_2_ convective dissolution. Thus, with increases in salinity, the convection starting time delay, the length of fingers, and fingers growth rate decrease.
3. Although increasing the temperature causes an increase in the diffusion coefficient, the solubility of CO_2_ in water decreases the density difference. An increase in temperature leads to a reduction in the initial wave number and CO_2_ affected area, which can adversely affect CO_2_ dissolution.
4. The spectrophotometric method was successfully used to quantitatively measure the dissolved CO_2_ concentration. The mass of dissolved CO_2_ due to convective dissolution is much greater than that for diffusion dissolution. The dimensionless flux, where the Sherwood number shows a power-law relationship with the Rayleigh number, indicates that the *Sh* increases with increasing *Ra*, and this refers to the lower salinity and temperature increasing the mass transfer flux of CO_2_ dissolution.
5. Salinity and temperature effects on convective instability of numerical simulation are in qualitative agreement with the experimental result. The shape and position of the convective finger in the simulation are influenced by the assumption of boundary conditions. Numerical simulation of CO_2_ convective dissolution still requires further research.

A quantitative and visualization study of the CO_2_ dissolution process is important for the further development of technology and simulation models. A determination of the dissolution flux can help to estimate the amount of CO_2_ that will remain in brine during the CO_2_ storage in saline aquifers. Our findings improve the understanding of the CO_2_ dissolution mechanism and help to assess the CO_2_ solubility trapping rate.

## Figures and Tables

**Table 1:** Properties of experimental fluids.

No.	Temperature (°C)	Salinity (mg/L)	∆*ρ* (kg/m^3^)	Viscosity (kg/m s)	Diffusion Coefficient (m^2^/s)	Rayleigh Number
1	25	0	0.37	9.13 × 10^−4^	1.85 × 10^−9^	17,406.23
2	25	5000	0.36	9.27 × 10^−4^	1.85 × 10^−9^	17,143.36
3	25	10,000	0.35	9.42 × 10^−4^	1.85 × 10^−9^	16,401.75
4	25	15,000	0.34	1.03 × 10^−3^	1.85 × 10^−9^	15,362.34
5	35	0	0.25	7.08 × 10^−4^	2.18 × 10^−9^	13,228.03
6	45	0	0.19	5.94 × 10^−4^	3.03 × 10^−9^	8621.23

**Table 2:** Extreme absorbance values and p*K_HIn_* at different temperatures by experiments.

	25 °C	35 °C	45 °C
*A*_HIn_	0.039	0.044	0.051
*A*_In-_	0.987	0.993	1.014
p*K_HIn_*	4.67	4.71	4.77

## Data Availability

Data sharing not applicable.
